# Supplementary material for: Tailoring Mesopores and Nitrogen Groups of Carbon Nanofibers for Polysulfide Entrapment in Lithium–Sulfur Batteries
Source: Polymers (Basel). 2022 Mar 25;14(7):1342. doi: 10.3390/polym14071342 (PMC9002479; doi:10.3390/polym14071342)
Supplement: Supplementary file 1 [file polymers-14-01342-s001.zip › polymers-1639733-supplementary.pdf]

Supplementary Materials

Tailoring Mesopores and Nitrogen Groups of Carbon Nano-fibers for Polysulfide Entrapment in Lithium-Sulfur Batteries

Snatika Sarkar[1], Jong Sung Won[1], Meichun An[1] Rui Zhang[1], Jin Hong Lee[1], Seung-Goo Lee[2], and Yong Lak Joo[1,*]

[1] Robert Frederick Smith School of Chemical and Biomolecular Engineering, Cornell University, Ithaca, NY, 14853, USA
[2] Department of Advanced Organic Materials & Textile Engineering, Chungnam National University, Daejeon 34134, Republic of Korea
Corresponding Author: Email: ylj2@cornell.edu (Y.L Joo)
Tel: +1-607-55-8591 (Y. L Joo)

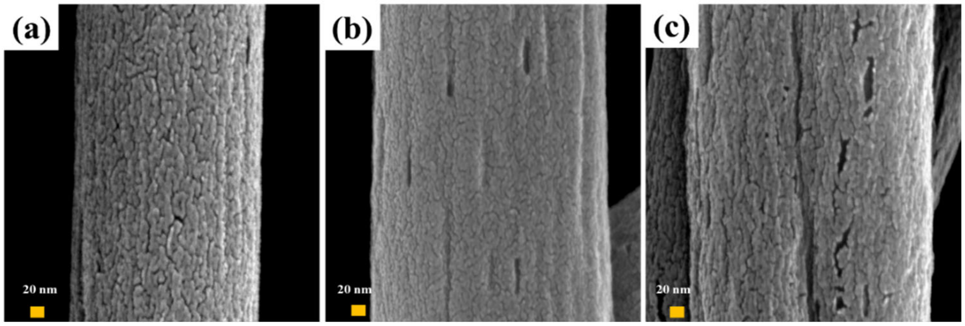

Figure S1. SEM surface images of carbon nanofibers obtained with different weight ratio at ceramic pressure load of 1.28 N: (a) PAN/PMMA (56:44); (b) PAN/PMMA (77:23); (c) PAN/PMMA (80:20).

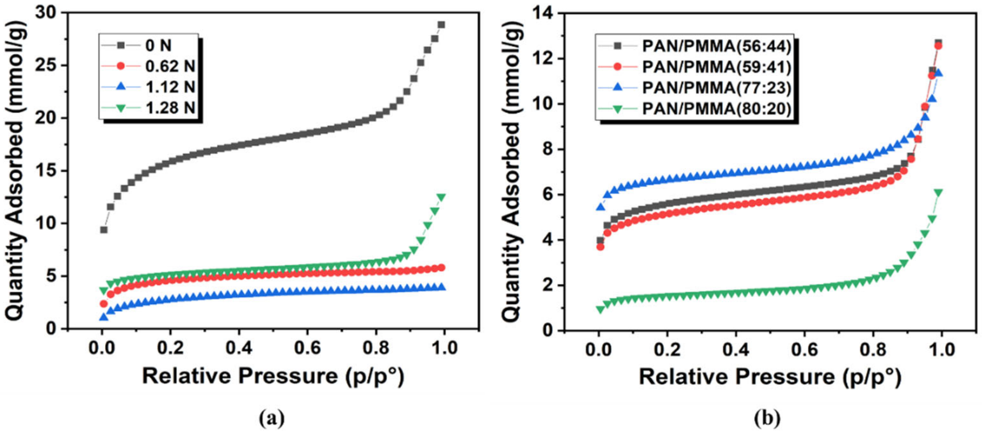

Figure S2. N_2 adsorption-desorption isotherms of carbon nanofibers prepared with different PAN/PMMA weight ratios and ceramic pressure loads.

Having confirmed the cyclization of mpCNF, the effects of CPAN on the electrochemical performance of the cells was tested by using them as both cathodes and interlayers in the following combinations of systems:

- mpCNF cathode and mpCNF interlayer (mpCNF-mpCNF)
- mpCNF cathode and CPAN interlayer (mpCNF-CPAN)
- CPAN cathode and mpCNF interlayer (CPAN-mpCNF)
- CPAN cathode and CPAN interlayer (CPAN-CPAN)

Cycle performances were carried out at 0.25C for 100 cycles using sulfur loadings 1.1mg/cm^2 and 1.7mg/cm^2.

As seen in Fig S3 and Fig S4, for both the sulfur loadings, we find that system mpCNF-CPAN displays highest capacity and best capacity retention. Interestingly, system CPAN-CPAN has the lowest capacity, performing poorly compared to system mpCNF-mpCNF which has no nitrogen functional groups. Rate capability tests of the above systems were compared at both loadings. Rate capability tests indicate that using CPAN as cathode may not be suitable for higher Sulfur loadings and higher C-rates.

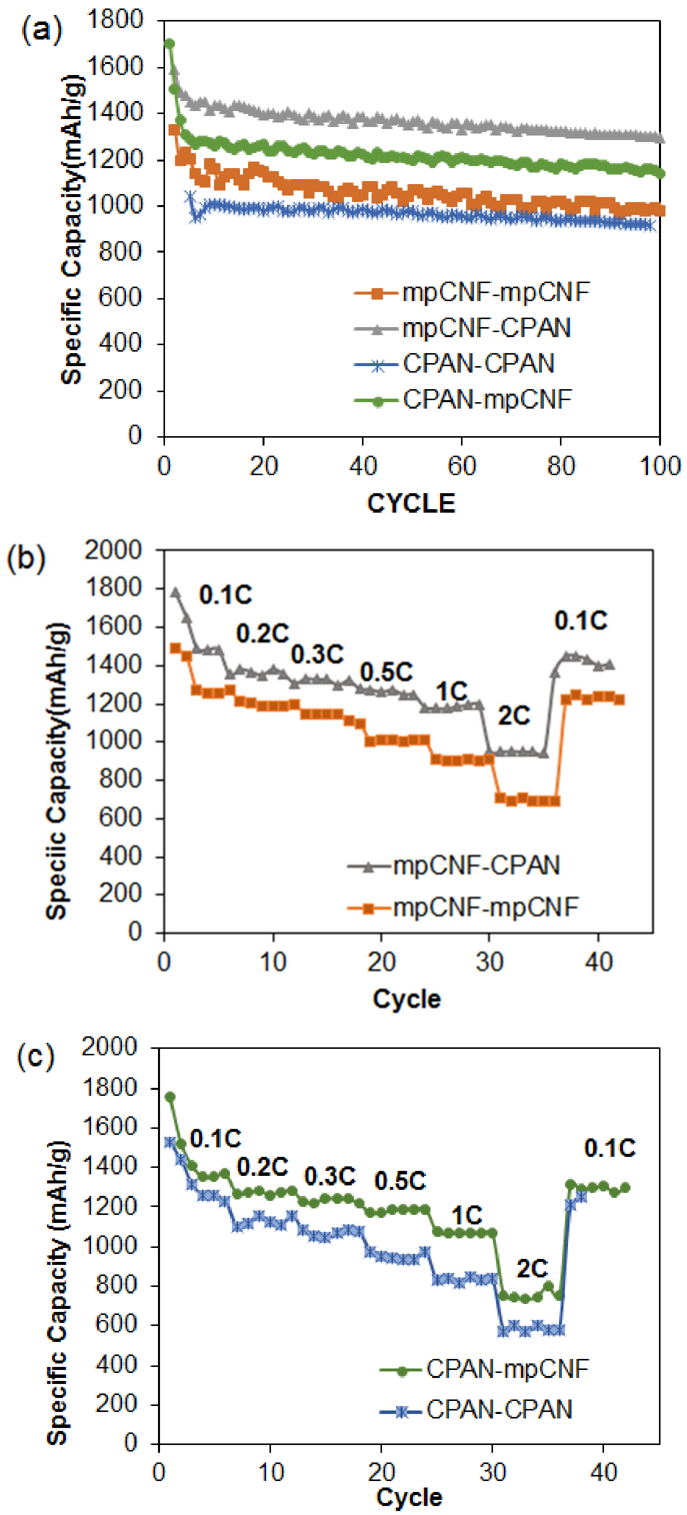

Figure S3. (a) Cycling performance of 4 systems at 1.1 mg/cm2 S-loading, 0.25C; (b) Rate Capability test comparison of systems using CPAN as interlayer at 1.1 mg/cm2 S-loading; (c) Rate Capability test comparison of systems using CPAN as cathode at 1.1 mg/cm2 S-loading.

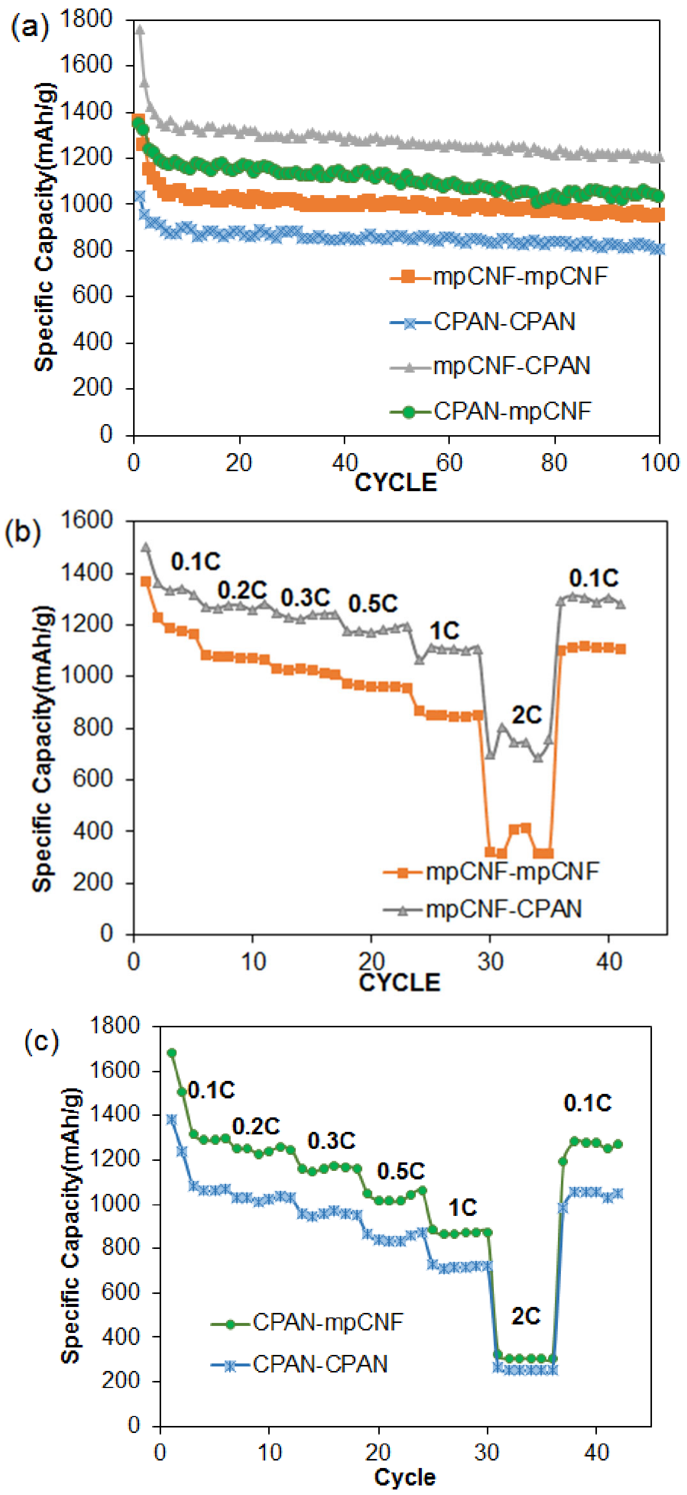

Figure S4. (a) Cycling performance of 4 systems at 1.7 mg/cm2 S-loading, 0.25C; (b) Rate Capability test comparison of systems using CPAN as interlayer at 1.7 mg/cm2 S-loading; (c) Rate Capability test comparison of systems using CPAN as cathode at 1.7 mg/cm2 S-loading.

Electrochemical Impedance Spectroscopy (EIS) of fresh cells are shown in Fig S5. Fresh cells, except the CPAN-CPAN system show comparable R_{ct} values indicating similar conductivity. However, CPAN-CPAN system has higher R_{ct} values compared to the remaining 3 systems. EIS spectra of each system after 100 cycles were also compared, as shown in Fig S5(c), (d). To compare the polysulfide reutilization capability of each system, the cells were charged to 2.8V before measuring the EIS spectra. It was observed that systems mpCNF-CPAN and CPAN-mpCNF showed a greater reduction in R_{ct} after 100 cycles. More importantly, they retained one semicircle even after charging as opposed to mpCNF-mpCNF and CPAN-CPAN systems. In the latter 2 systems, a second semicircle emerged in the high frequency region, which is attributed to increased surface impedance due to formation of Li_2S precipitate film on the cathode surface [27,28]. This indicates that during the charge cycle, not all the polysulfides were converted back to Sulfur, implying poor Sulfur reutilization capability of mpCNF-mpCNF and CPAN-CPAN systems

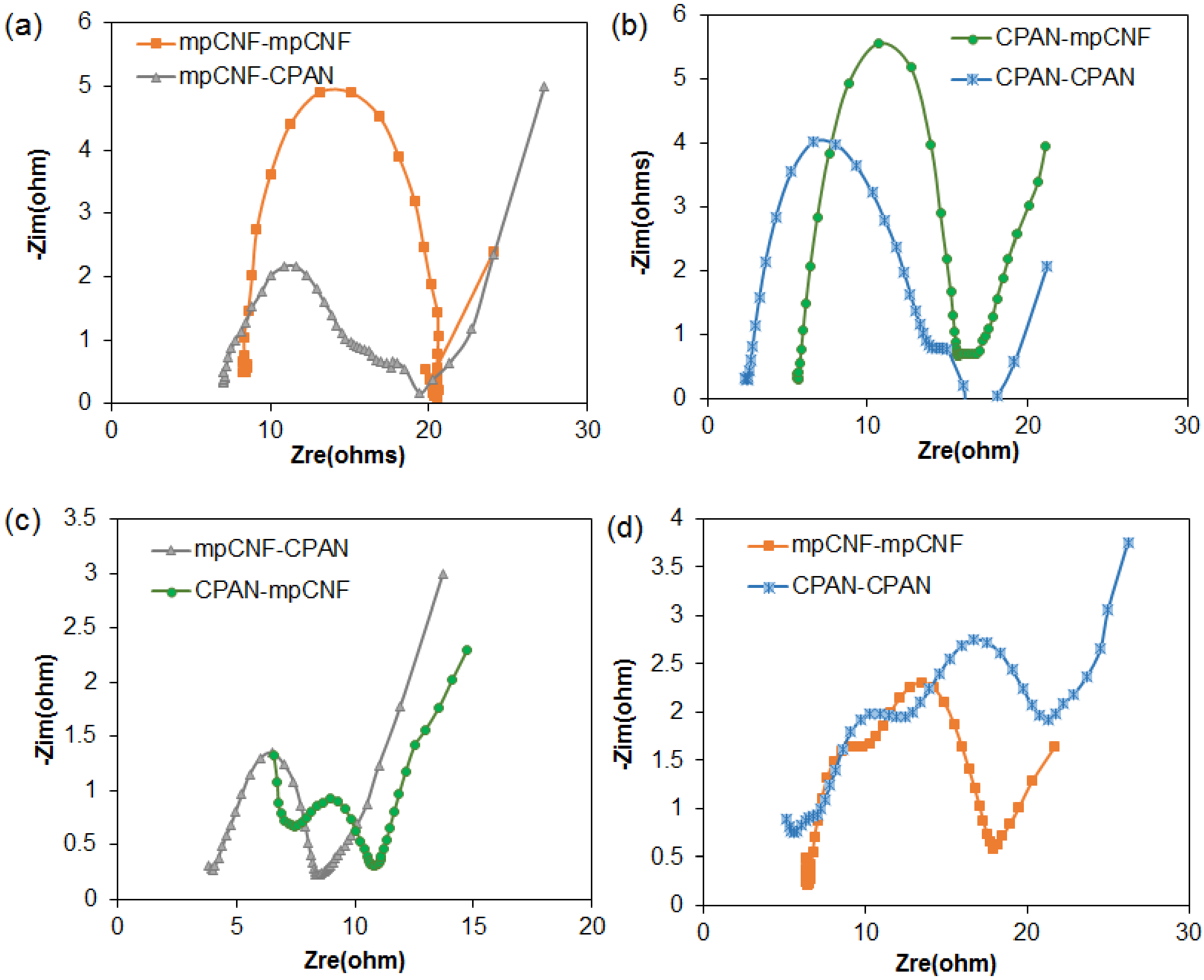

Figure S5. EIS Spectra of fresh cells (a) mpCNF-mpCNF and mpCNF-CPAN; (b) CPAN-mpCNF and CPAN-CPAN EIS Spectra of discharged cells after 100 cycles; (c) mpCNF-CPAN and CPAN-mpCNF; (d) mpCNF-mpCNF and CPAN-CPAN.

High resolution S 2*p* spectra of the cathode and interlayer of each system was examined after 100 cycles and are shown in Fig S6 (a)-(d). The peak at high binding energy appeared in Fig S6 is attributed to oxidized sulfur species such as sulfite $[SO_3]^{2-}$ at 167 eV, or sulfate $[SO_4]^{2-}$ at 169 eV, while the peak at the low binding energy region is assigned to sulfides such as Li_2S at 160 eV. The higher intensity of the polysulfide peak in CPAN cathodes

and interlayers confirms that their polysulfide reutilization capability is better than that of mpCNF. Moreover, The Li_2S signals are significantly weaker in the CPAN cathode as opposed to CPAN interlayer. This implies that despite the presence of Nitrogen functional groups utilizing CPAN as a cathode results in greater active material loss than mpCNF cathode, possibly due to the lack of mesopores.

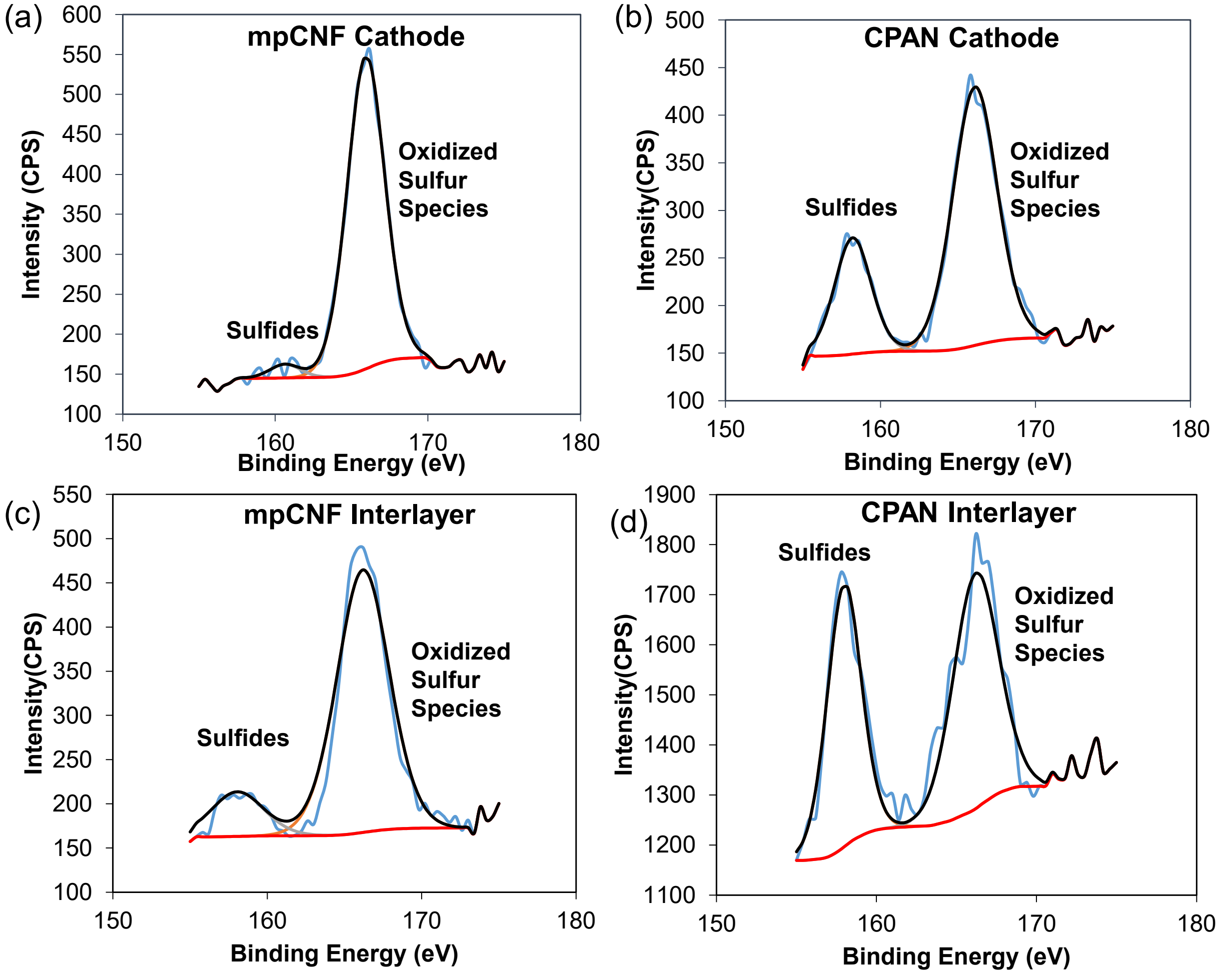

Figure S6. High Resolution S 2p spectra of (a) mpCNF cathode; (b) CPAN cathode; (c) mpCNF interlayer; (d) CPAN interlayer.

To further confirm the effectiveness of the Nitrogen groups in capturing polysulfides, high resolution N 1s spectra of systems utilizing CPAN as cathode or interlayer or both, were examined in Fig S7. In the case of mpCNF-CPAN, the N 1s spectra of the interlayer was deconvoluted to obtain 2 peaks at 399.2 and 397eV. However, it was observed that the shifted peak corresponding to Li-N interactions in CPAN interlayer has a higher intensity and larger area than the peak for cyclic nitrogen groups. This indicates that the interlayer was not the reason behind lower capacity of CPAN-CPAN. Interestingly, for the case of CPAN cathodes, peak deconvolution showed 2 peaks corresponding to Pyridinic Nitrogen and a shifted peak to a binding energy of 395.2 eV. The peak for Pyridinic Nitrogen had a much larger area and Intensity than the shifted peak, implying that using CPAN as cathode results in poorer Nitrogen group utilization for polysulfide capture. This could explain the poorer cycle performance of CPAN-CPAN relative to the other systems.

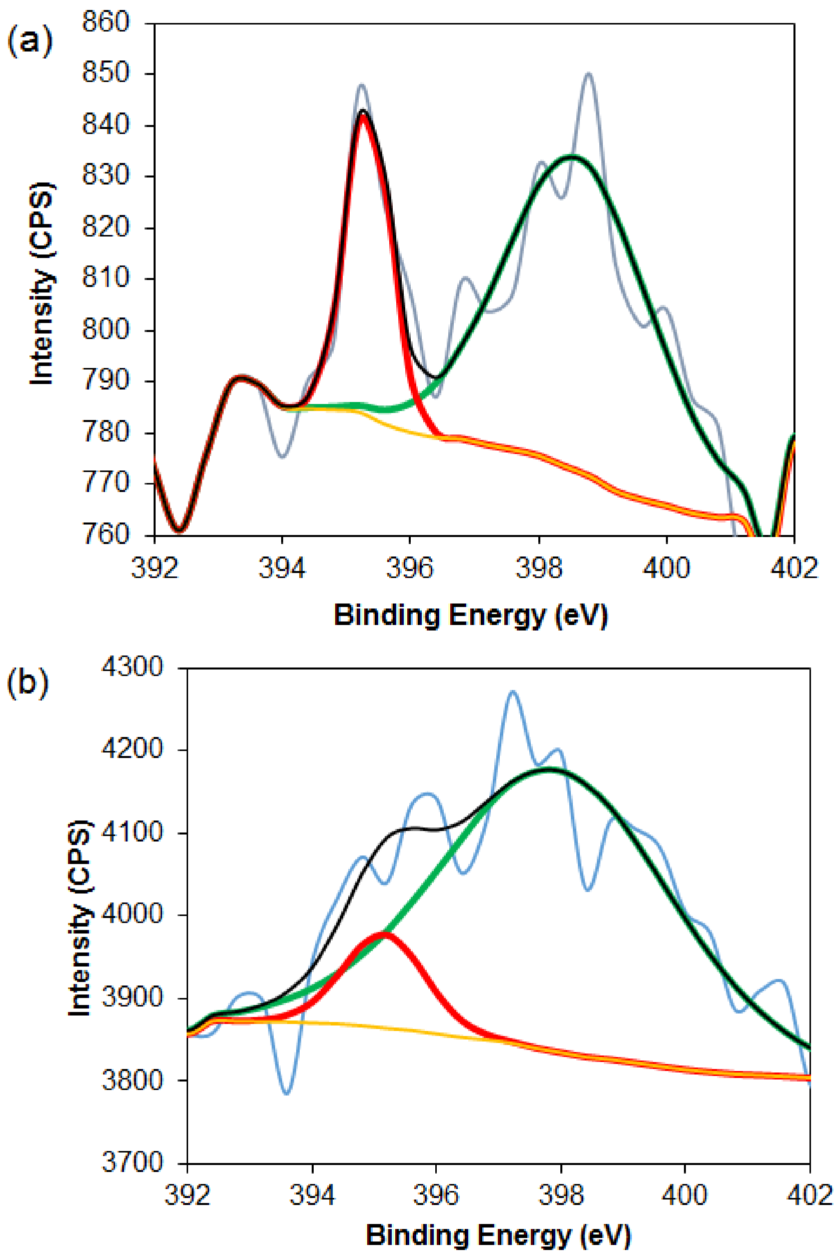

Figure S7. High Resolution N 1s spectra of (a) CPAN interlayer (b) CPAN cathode

The significant reduction in surface area and loss of pores in CPAN indicates lower active sites available for Sulfur deposition and electrochemical reactions. This explains why CPAN, with a high N-content but low mesopore volume performs better as an interlayer

than a cathode. Sulfur, which was incorporated into the cathode by electrospraying, may have deposited only on the surface of CPAN, instead of being trapped in the active pores as it does in mpCNF. This may have resulted in eventual loss of active material over several cycles. Moreover, the reduced surface area can reduce the Lithium Sulfide storage capability and slow down ion diffusion due to increased tortuosity. In the 4 CPAN systems studied, CPAN-CPAN was the only one that did not have any component with a large surface area and mesopores/micropores. In the remaining 3 systems, there was at least 1 component that had the high surface area required for electrochemical reactions, and therefore they showed a better cycle performance than CPAN-CPAN.
